# Insights into the Silylation of Benzodiazepines Using *N*,*O*-Bis(trimethylsilyl)trifluoroacetamide (BSTFA): In Search of Optimal Conditions for Forensic Analysis by GC-MS

**DOI:** 10.3390/molecules29245884

**Published:** 2024-12-13

**Authors:** Eleazar Vargas Mena, Eliana R. Herrera Giraldo, Jovanny A. Gómez Castaño

**Affiliations:** 1 Grupo Ciencias Forenses, Laboratorio de Toxicología Regional de Occidente, Instituto Nacional de Medicina Legal y Ciencias Forenses, Avenida de las Américas No 98-25, Pereira 660004, Colombia; 2 Grupo Química-Física Molecular y Modelamiento Computacional (QUIMOL), Escuela de Ciencias Químicas, Universidad Pedagógica y Tecnológica de Colombia, Sede Tunja, Avenida Central del Norte, Boyacá 150003, Colombia; grupo.quimol@uptc.edu.co

**Keywords:** benzodiazepine analysis, forensic toxicology, gas chromatography-mass spectrometry, silylation derivatization, designer benzodiazepines, BSTFA

## Abstract

Silylation is a widely used derivatization technique for the gas chromatographic analysis of benzodiazepines, a class of psychoactive drugs commonly encountered in forensic and biological samples. This study investigated the optimal experimental conditions for the silylation of benzodiazepines using *N*,*O*-bis(trimethylsilyl)trifluoroacetamide containing 1% trimethylchlorosilane (BSTFA + 1% TMCS), a widely employed silylating agent. Ten structurally different benzodiazepines, including variations within the classic 1,4-benzodiazepine core and triazolo ring derivatives, were selected to address the effect of structural diversity on silylation. Principal component analysis (PCA) and hierarchical cluster analysis (HCA) were used to optimize the silylation of benzodiazepines by means of GC-MS analysis. PCA identified key experimental factors influencing silylation efficiency and distinct response patterns of different benzodiazepines. HCA further categorized the benzodiazepines based on their silylation behavior, highlighting the need for tailored derivatization strategies. The results indicated that the BSTFA + 1% TMCS concentration and solvent volume were pivotal for achieving high silylation efficiency, whereas the temperature, reaction time, and catalyst were less critical. The optimized method was successfully applied to 30 real forensic samples, demonstrating its efficacy in detecting and identifying various benzodiazepines, including designer drugs like etizolam. This study provides a foundation for improving drug detection methodologies in forensic toxicology and provides useful insights into the dynamics of benzodiazepine silylation and the use of individualized analysis parameters.

## 1. Introduction

Benzodiazepines are a widely used class of depressant drugs that are often referred to as “downers” [1]. They have sedative, hypnotic, anxiolytic, amnestic, anticonvulsant, and muscle relaxant properties and are prescribed to treat various central nervous system (CNS) disorders such as anxiety, depression, insomnia, seizures, and epilepsy, as well as spasms and alcohol withdrawal cases [2]. The action of benzodiazepines on the CNS occurs by enhancing the effect of the endogenous neurotransmitter gamma-aminobutyric acid (GABA) on the GABAA receptor through indirect inhibitory modulation by binding to a peripheral allosteric site known as the benzodiazepine site [3,4]. Although benzodiazepines are generally well tolerated and effective for short-term use in treating several conditions, their high psychotropic activity makes them commonly associated with violence, intoxications, and accidents, including drug abuse, sexual assaults, thefts, accidental deaths, suicides, homicides, among other issues [5,6].

**Figure 1:**
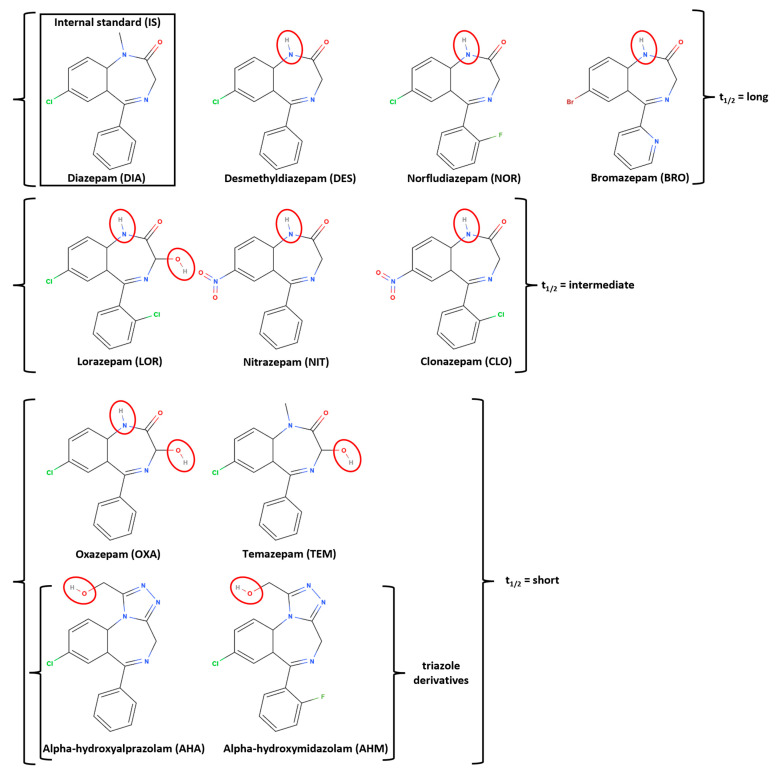
Structural formula images of the benzodiazepines investigated in this study. The functional groups prone to silylation with BSTFA + 1% TMCS are indicated by red ovals. Structures are grouped in brackets according to their metabolic elimination half-life (t_1⁄2_) range. The 3-letter abbreviations in parentheses are used to refer to the selected benzodiazepines henceforth.

The core structural feature of benzodiazepines is the presence of a benzene ring fused to a diazepine ring (Figure 1) [7]. These compounds can be classified based on their substitutions as 2-keto, 3-hydroxy, 7-nitro, triazolo, or imidazo derivatives. Another classification system for benzodiazepines comes from their metabolic elimination half-life range (t_1⁄2_), allowing them to be categorized as short-acting (1 to 12 h), intermediate-acting (12 to 40 h), or long-acting (40 to 250 h) active metabolites [8,9].

**Scheme 1:**
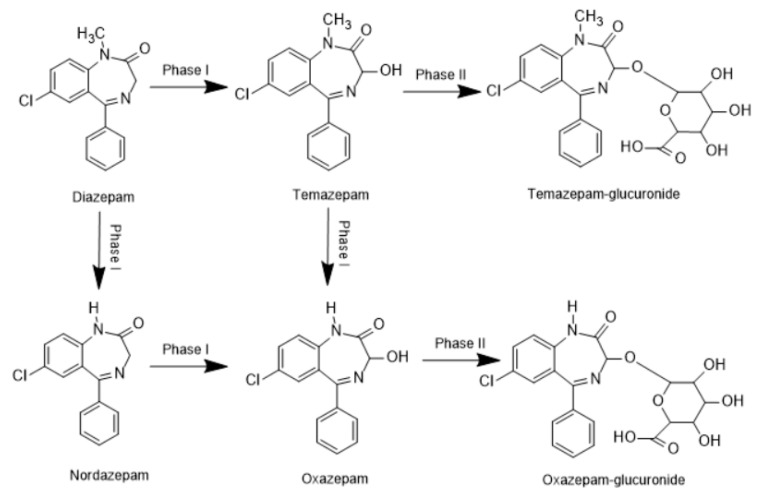
The metabolic pathways of diazepam.

Most benzodiazepines are orally administered and reach peak blood levels (as protein complexes) approximately 1 h after absorption. Some may also be administered intravenously, intramuscularly, or rectally. The metabolism of benzodiazepines is complex because of the in situ production of other benzodiazepine intermediates. They can be categorized into two main hepatic metabolic pathways. In phase I biotransformation, they undergo extensive cytochrome P450 isoenzyme-mediated metabolism, increasing the polarity of the active metabolite through sequential reactions (mainly oxidation and hydroxylation). Phase II consists of the covalent addition of glucuronic acid via *N*-glucuronidation or *O*-glucuronidation of the parent compound, catalyzed by UDP-glucuronosyltransferase (UGTS) enzymes, producing hydrophilic, water-soluble, and conjugated metabolites [10]. In both cases, due to their polarity, most benzodiazepine metabolites are primarily eliminated in the bile and urine [11]. For example, the benzodiazepine diazepam, a commonly studied compound, undergoes extensive metabolism primarily mediated by the cytochrome P450 enzymes CYP3A4 and CYP2C19, producing the active metabolites nordiazepam, temazepam, and oxazepam [11], as shown in Scheme 1.

Routine toxicological analysis of benzodiazepines in patients typically involves enzymatic treatment of urine samples to restore the original molecular structure of the benzodiazepine precursor [10]. These urine samples are then subjected to immunoassay screening using methodologies such as the enzyme-multiplied immunoassay technique (EMIT^®^), enzyme-linked immunosorbent assay (ELISA), or radioimmunoassay (RIA). Confirmatory analyses are mainly conducted by high-performance liquid chromatography coupled with tandem mass spectrometry (HPLC-MS, HPLC-MS/MS, and UHPLC-MS/MS) or gas chromatography coupled with single or tandem mass spectrometry configurations (GC-MS, GC-MS/MS) [12,13].

Urine samples are preferred for benzodiazepine analysis because they are noninvasive and easily accessible. These matrices also have high-volume capability and a longer detection window than other matrices, such as blood or saliva [10]. Urine is frequently chosen as the analysis matrix owing to its high availability as the primary forensic protocol sample and its compatibility with immunoassay techniques, enabling targeted confirmatory analysis. Blood and, to a lesser extent, bile and vitreous humor are used to confirm impairment and quantify results previously obtained from urine. Analyzing urine offers several advantages, such as saving resources and reducing the analytical response time.

Restored benzodiazepine structures in urine samples are commonly derivatized using suitable silylating reagents, such as *N*,*O*-bis(trimethylsilyl)trifluoroacetamide (BSTFA), to improve their detectability in analytical methods [14]. Appropriate silylation methodologies are crucial for enhancing the detection and quantification of benzodiazepines and their metabolites [15]. Silylation with trialkylsilyl-type reagents, including BSTFA, has been widely used because of the abundance of reported mass spectra of the trimethylsilated derivatives [16]. Other derivatization methodologies, such as propylation, propionylation, and acylation, have also been reported [15]. All these methods aim to replace the hydrogens attached to the NH and OH groups with polar groups to form a more volatile and thermally stable structure suitable for GC-MS analysis.

Although LC-MS/MS has become the most widely used methodology for analyzing substances of forensic interest due to its wide chemical space coverage, the lack of libraries for high-resolution mass spectrometry remains a challenging step in identifying compounds related to high-resolution spectra. In contrast, GC-MS analysis offers advantages such as lower instrumental cost, better chromatographic resolution, and compound annotation capacity provided by the extensive coverage of electron-impact (EI) trimethylsilyl (TMS) forensic compound spectral libraries (e.g., NIST, Cayman).

The structural diversity of benzodiazepines and the range of derivatization reagents used to produce volatile products have made it challenging to establish optimal experimental conditions for reliable and reproducible quantification of these psychotropic substances in biological samples by GC-MS. Consequently, this study aimed to investigate the best experimental conditions for the silylation reaction of benzodiazepines using BSTFA + 1% TMCS as a silylating agent [17] and to evaluate the effects of these conditions on the volatilization and stability of the compounds during GC-MS analysis.

The methodological design consisted of two rounds. The first round evaluated the effects of reaction time, temperature, amount of derivatizing reagent, and solvent on eight benzodiazepines. The second round kept significant conditions from the first round as variables while including the effects of pyridine as a catalyst and acetonitrile as a second solvent on 10 benzodiazepines [18]. Figure 1 shows the chemical structures of the benzodiazepines investigated in this study. The experimental conditions in both rounds were established using a Box–Behnken-type factorial design, which offers advantages over other factorial designs [19]. The silylation response was quantified using the relative response factor (*RRF*), i.e., the ratio of the analyte signal to the internal standard (IS) signal, to optimize the silylation process. The findings of this study provide valuable insights into the optimal experimental conditions for the silylation of benzodiazepines using BSTFA + 1% TMCS, which can significantly enhance the detection and quantification of these psychotropic substances in biological samples when analyzed by GC-MS.

## 2. Results and Discussion

### 2.1. Initial Chemometric Evaluation: Exploring Main Silylation Conditions

#### 2.1.1. Dataset Overview

Table 1 shows the normalized *RRF* values for the 27 different experimental combinations of reaction conditions tested in the study of the silylation reaction of the benzodiazepines LOR, OXA, BRO, TEM, NOR, AHM, DES, and AHA using BSTFA + 1% TMCS. This initial screening produced a wide range of *RRF* values across the four different tested experimental conditions (i.e., BSTFA + 1% TMCS concentration, ethyl acetate (EA) volume, reaction time, and temperature), indicating significant variations in the efficiency of the silylation reaction for the selected benzodiazepines. These results indicate that the selection of experimental conditions can greatly affect the silylation reaction efficiency of BSTFA + 1% TMCS.

The results presented in Table 1 indicate that only experiments 4–6, 21, and 24 had a positive silylation response across the entire set of analyzed benzodiazepines. This highlights BSTFA + 1% TMCS’s ability to consistently silylate benzodiazepines bearing diverse substituents and different structural characteristics under certain reaction conditions. These experiments produced the highest overall *RRF* values in this evaluation stage. The volume of BSTFA + 1% TMCS used was either 25 µL or 50 µL, while the reaction time ranged from 5 to 32.5 min. Interestingly, four of the five experiments (i.e., exp. 4–6 and 21) involved an initial addition of 25 µL of ethyl acetate (EA) as an anhydrous solvent; however, none required the maximum reaction time (i.e., 60 min) to achieve a positive silylation response. The temperature range of these five reactions encompassed all three available temperatures (30, 60, and 90 °C). This demonstrates that benzodiazepines can be effectively silylated using BSTFA + 1% TMCS at different temperatures within a relatively short timeframe (t < 32.5 min) [16,20].

Experiment 1 was the only trial at this stage that showed no silylation response across all benzodiazepines tested. This experiment combined a large volume of EA solvent (50 µL) with a minimal volume of BSTFA + 1% TMCS (25 µL) and the shortest reaction time (5 min). Similarly, other experiments involving 50 µL of EA and 25 µL of BSTFA + 1% TMCS (e.g., exp. 7 and 13–14) also exhibited low silylation responses across the series. The reduced silylation response with high EA volumes may be attributed to factors such as the dilution of the silylating agent BSTFA, which leads to decreased efficacy, as well as an imbalance between the solvent and silylating agent, which could obstruct the necessary interactions for silylation. Additionally, the excessive volume and properties of the solvent may have hindered BSTFA’s access to the reactive sites of benzodiazepines, further lowering its effect on the silylation process [21].

Overall, experiments 6 and 24 consistently exhibited the highest silylation responses across the benzodiazepine series. Experiment 6, using 25 µL of EA, 25 µL of BSTFA + 1% TMCS, and a 5 min reaction time at 90 °C produced high *RRF* values for all analytes. In contrast, experiment 24, with 50 µL of EA, 50 µL of BSTFA + 1% TMCS, and a 32.5 min reaction time at 60 °C, resulted in higher silylation responses for BRO and NOR. These findings underscore the complex interplay of factors influencing the silylation efficiency of BSTFA + 1% TMCS in benzodiazepines, including the balance between solvent volume and silylating agent quantity and their relationship with reaction time and temperature.

#### 2.1.2. Multivariate Analysis Using PCA

**Figure 2:**
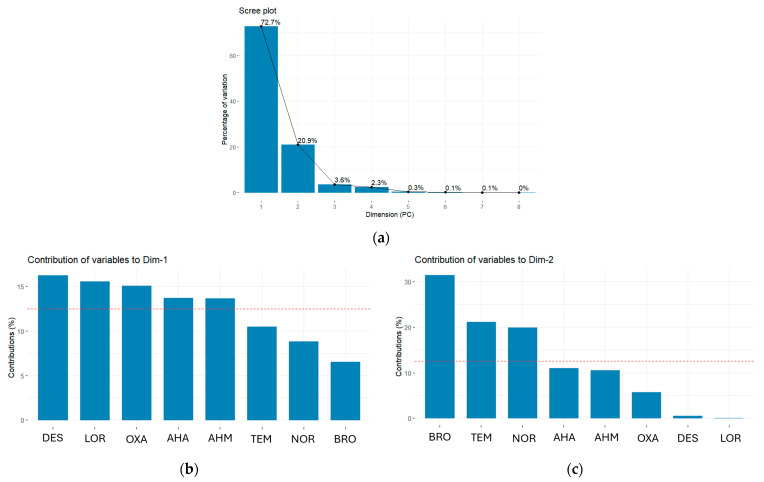
Scree plot (**a**) and plots of the contributions of variables to dimensions 1 and 2, (**b**,**c**), respectively, obtained from PCA for the first silylation panel of reaction conditions using BSTFA + 1% TMCS as the silylating agent. The dotted red line in graphs (**b**,**c**) indicates the mean value.

**Figure 3:**
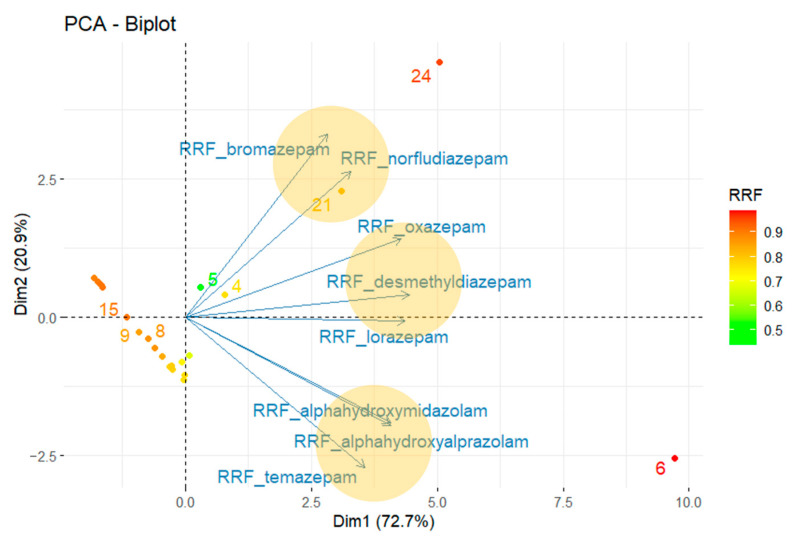
Scores biplot and distinct clusters derived from PCA of the first silylation panel for reaction conditions using BSTFA + 1% TMCS as the silylating agent. Clusters are indicated by orange circles.

To gain deeper insights into the experimental conditions and explore the underlying trends or patterns that produce the most efficient silylation of benzodiazepines with BSTFA + 1% TMCS, principal component analysis (PCA) was performed on the normalized *RRF* values. The PCA results of the first experimental evaluation round are presented in Figure 2 and Figure 3.

- Principal Components and Loadings

As shown in Figure 2a, the first two principal components account for 93.6% of the total variance, with PC1 representing 72.7% and PC2 capturing 20.9%. This indicates that most variance in the dataset can be explained by analyzing only these two principal components. The high variance explained by PC1 reveals that most silylation response factors were significantly influenced by the reaction conditions (i.e., BSTFA + 1% TMCS volume, EA volume, reaction temperature, and reaction time). Variables with high variability, such as BSTFA + 1% TMCS volume and EA volume, likely contribute the most to this principal component. PC2, on the other hand, is influenced by secondary factors that are not as dominant as those affecting PC1. These secondary factors can include specific interactions between the reaction conditions and chemical or structural properties (e.g., functional groups and substituents) of the tested benzodiazepines.

The bar charts presented in Figure 2b,c depict the loadings, i.e., the contributions of the different variables (benzodiazepines) to PC1 and PC2, respectively. DES, LOR, and OXA exhibited the highest positive contributions (above 15% each) to PC1, indicating that their silylation responses were strongly influenced by the primary reaction conditions. The contributions of approximately 13% of the triazole derivatives AHA and AHM to PC1 indicate that these compounds also play significant roles in the silylation process. Their OH groups might be particularly reactive under the tested conditions, leading to high *RRF* values. TEM and NOR contributed more moderately to PC1 (around 10%), suggesting that their silylation was affected by the reaction conditions to a lesser extent. The lower contribution of BRO (around 6%) indicates that it has the least impact on the variance captured by PC1. This might be due to the chemical structure of the compound being less reactive or more stable under the tested silylation conditions, resulting in lower *RRF* values.

The contribution graph for PC2 (Figure 2c) shows an almost opposite pattern to that of PC1. BRO exhibits the highest positive contribution to PC2 (around 30%), implying that its silylation response is significantly influenced by secondary factors not captured in PC1. This suggests that BRO’s silylation efficiency may be driven by factors different from the primary reaction conditions, possibly related to the presence of its bromine atom. TEM and NOR contributed approximately 20% each to PC2, indicating they are strongly influenced by the secondary factors represented in this component. This implies that their silylation behavior is affected by a combination of the primary reaction conditions and additional chemical or structural properties not considered in the first principal component. The triazole derivatives AHA and AHM contribute just over 10% to PC2, indicating a moderate influence from secondary factors. OXA contributes just over 5% to PC2, whereas DES and LOR make the lowest contributions to this component, below 1.0% each. Thus, the secondary factors captured by PC2 had a relatively minor impact on OXA, DES, and LOR silylation compared with the other benzodiazepines studied. The results suggest that both the chemical structure and steric hindrance of benzodiazepines can influence their silylation reaction when using BSTFA + 1% TMCS as the silylating agent, as evidenced by the varying contributions of the compounds to the principal components.

- PCA Biplot and Clustering Analysis

The PCA biplot presented in Figure 3 offers a visual depiction of the relationships between the benzodiazepine compounds and the two principal components derived from PCA. In this graph, the arrows represent the eigenvectors of each benzodiazepine, indicating both their direction and magnitude of contribution to each dimension. The eight eigenvectors are situated in the right quadrants of the biplot, in which experiments with consistently positive outcomes are located (i.e., exp. 4–6, 21 and 24). These eigenvectors exhibit similar magnitudes but differ in direction, indicating that the silylation reaction of benzodiazepines using BSTFA + 1% TMCS is influenced by multiple factors with the potential for both positive and negative results. Despite the radial distribution of eigenvectors, three main cluster patterns were identified in this PCA biplot.

The first cluster pattern includes TEM and the two triazole derivatives AHA and AHM. These compounds are closely grouped in the right lower quadrant and share similar contributions to PC1 and PC2. They exhibited the highest sensitivity in this trial of experiments. TEM displayed a positive silylation response in 24 out of 27 experiments (rate ~89%), whereas both AHA and AHM showed positive silylation responses in 25 out of 27 experiments (rate ~93%). These three benzodiazepines share the structural feature of having a single OH group suitable for silylation. This may explain their similar reaction behavior. Previous silylation experiments of TEM, AHA, and AHM with various silylating agents also showed high efficiency when using BSTFA + 1% TMCS compared with the other benzodiazepines tested in this study, except for NOR [16]. These results demonstrate that the presence of a unique OH group contributes to high sensitivity and a positive silylation response when BSTFA + 1% TMCS is used as a silylating agent. Additionally, TEM, AHA, and AHM consistently showed positive silylation responses even in experiments without the addition of BSTFA + 1% TMCS (i.e., experiments 3, 12, 18–19, 22, and 27). A plausible explanation for this response is the potential presence of control benzodiazepine (DIA) as an impurity or degradation product in these compounds under these experimental conditions.

LOR, OXA, and DES form the second cluster pattern in the PCA biplot. Their eigenvectors align closely with the positive coordinate axis of PC1. This cluster comprises benzodiazepines with intermediate-to-low silylation responses that exhibit both positive and negative results in different experiments. These benzodiazepines consist of three chlorinated derivatives with closely related structures. LOR and OXA differ only by the presence/absence of a second chlorine atom in the ortho position in the phenyl substituent, whereas DES differs from OXA only by changing OH to H in the diazepine ring. Notably, LOR and OXA are the only two structures of the series that share two substitutional groups (NH and OH) prone to silylation with BSTFA. LOR and OXA exhibited positive silylation responses only in the subset of five experiments (rate ~ 19%) that consistently produced a positive response throughout all trials (i.e., experiments 4–6, 21, and 24). In contrast, DER exhibited positive silylation reactions ten times when reacted with BSTFA + 1% TMCS (experiments: 4–6, 11, 16, 21, 23–26), indicating an approximate silylation efficiency of 37%. As silylation typically targets hydroxyl groups to convert them into trimethylsilyl ethers, the lower sensitivity of LOR and OXA to silylation may be due to the steric hindrance around their hydroxyl groups, which makes them less accessible for reaction with silylating agents.

The third cluster pattern in the PCA biplot comprises NOR and BRO. These two benzodiazepines showed silylation responses that were different from those of the other compounds studied. NOR, which is structurally related to DES, exhibited a positive response to specific reaction conditions (experiments 4–6, 11, 16, 21, 23–26), yielding an approximately 37% response. However, except for experiments 24 and 25, DES generally had higher *RRF* values than its fluorinated analog (NOR). The similarities between NOR and DES were evident in their low silylation activity under certain conditions; specifically, both compounds showed limited or no detectable activity in select experiments with identical variables. Notably, NOR produced the highest *RRF* value among all evaluated benzodiazepines (*RRF* = 9.86 in experiment 24), indicating high sensitivity to silylation under these specific reaction parameters (BSTFA + 1% TMCS and EA volume = 50 µL; reaction temperature = 60 °C; reaction time = 32.5 min). This result is consistent with the high sensitivity of NOR silylation using BSTFA + 1% TMCS, as previously reported [16]. Conversely, BRO exhibited the lowest overall silylation response among the evaluated benzodiazepines (0.058 ≥ *RRF* ≥ 0.309). The restricted reactivity of BRO can be attributed to the steric hindrance posed by its bulky pyridine and bromine substituents, along with the presence of only one NH group. This makes silylation less accessible than that of other benzodiazepines.

#### 2.1.3. ANOVA Boxplots

**Figure 4:**
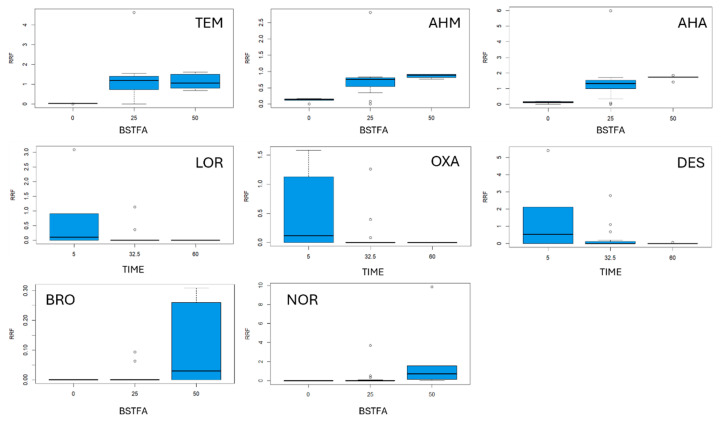
ANOVA boxplots showing the silylation responses of the eight benzodiazepines against the four experimental conditions studied.

ANOVA boxplots were used to assess and compare the silylation responses of the eight benzodiazepines across the four experimental conditions. Figure 4 shows specific ANOVA boxplots linked to variables with statistical significance (*p* < 0.05). The statistical data from the ANOVA analysis for each benzodiazepine are summarized in the Supplementary Material.

The top row in Figure 4 displays the boxplots for the cluster formed by the benzodiazepines TEM, AHA, and AHM. It is evident that the silylation of these benzodiazepines was primarily affected by the amount of BSTFA + 1% TMCS used in the reaction, with higher volumes leading to increased silylation responses. Additionally, the reaction time significantly affected the silylation of these benzodiazepines. However, with less than the BSTFA + 1% TMCS volume, longer reaction times resulted in slightly higher silylation responses. These results demonstrate that the BSTFA + 1% TMCS concentration significantly affects the silylation of TEM, AHA, and AHM owing to the accelerated reaction rate resulting from increased collisions. Furthermore, longer reaction times result in more thorough silylation of these benzodiazepines.

The middle row in Figure 4 shows the boxplots for the benzodiazepine cluster formed by LOR, OXA, and DES. The silylation activity of these derivatives was significantly affected by the reaction time, with the highest distribution of *RRF* values observed at a shorter time (5 min). This result indicates that shorter reaction times resulted in higher silylation responses for LOR, OXA, and DES. In the case of OXA, the amount of BSTFA + 1% TMCS also had a noticeable effect (*p* ≈ 0.03) on the silylation response; higher volumes resulted in higher *RRF* values.

The bottom row in Figure 4 groups the boxplots for the benzodiazepines BRO and NOR. The silylation of BRO and NOR was less efficient than that of the other benzodiazepines. Higher BSTFA + 1% TMCS volumes resulted in higher silylation responses, indicating that the BSTFA + 1% TMCS concentration was a key factor affecting the silylation reaction in these structures.

### 2.2. Second Chemometric Evaluation: Refining Silylation Conditions

The objective of the second evaluation panel for silylation conditions was to further optimize experimental parameters for the derivatization reaction of benzodiazepines using BSTFA + 1% TMCS as a silylating agent. In stage 1, a correlation was observed between the BSTFA + 1% TMCS concentration and EA volume, whereas time and temperature showed no consistent effect. Therefore, in this second stage of evaluation, the maximum amount of BSTFA + 1% TMCS was used (50 µL) for all experiments, with EA volume, reaction time, and temperature as variables. Additionally, acetonitrile (ACN) was introduced as an alternative anhydrous solvent, and the addition of pyridine was evaluated as a potential catalytic agent. In addition, two new compounds were included: CLO and NIT, whereas OXA-d5 was used as the standard. The dataset of standardized *RRF* values obtained from the second evaluation is presented in Table 2.

#### 2.2.1. Dataset Overview

In the second evaluation panel, 8 out of 46 experiments (experiments 3–4, 10–11, 14, 33, 36, and 46) produced positive silylation responses for all benzodiazepines tested. These were the only cases in which a positive silylation reaction was observed for nitrated benzodiazepines (i.e., CLO and NIT). Six of these trials were conducted at 60 °C with a reaction time of 5 min. The remaining two trials (experiments 36 and 46) were conducted at 90 °C for 5 min and 30 °C for 32.5 min, respectively. Seven of the eight experiments required the addition of pyridine as the catalytic agent. Experiment 3 was the only case with positive silylation responses without the use of pyridine. All possible combinations of mixtures between EA and ACN solvents were used in the eight experiments, resulting in consistently positive silylation responses.

Experiment 24, conducted at 60 °C for 32.5 min using ACN as the sole solvent and adding 25 µL of pyridine, displayed remarkable *RRF* values for all the non-nitrated benzodiazepines tested. This was the last test in which a positive silylation response to LOR was observed, excluding the eight experiments mentioned earlier.

Experiments 1, 2, 16, 32, and 42 resulted in high *RRF* values for OXA, TEM, AHM, NOR, DES, and AHA derivatives, but no silylation activity was observed for the other benzodiazepines. These experiments had the common condition of a temperature of 60 °C and a reaction time of 32.5 min. No evident systematic tendency in other variables was observed for these experiments. Surprisingly, experiment 32 was the only test in the entire evaluation that was performed without the use of solvents, yet it yielded high *RRF* values for specific benzodiazepine derivatives.

#### 2.2.2. Principal Component Analysis

**Figure 5:**
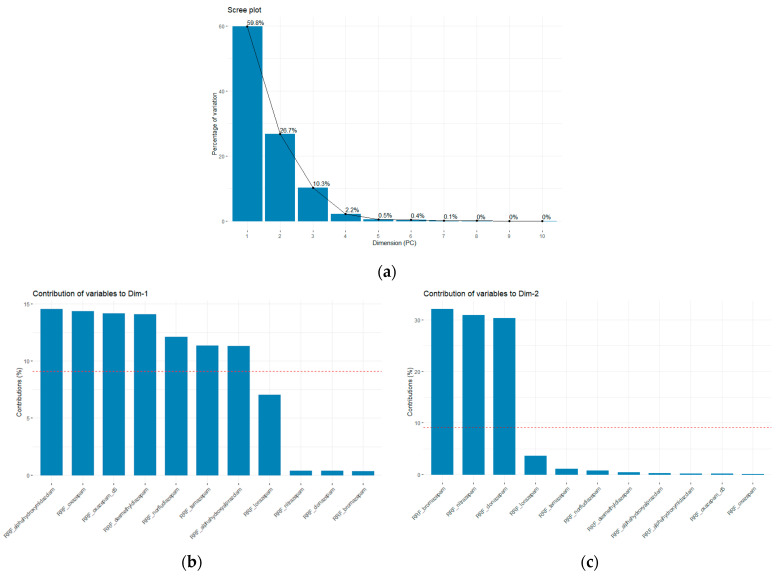
Scree plot (**a**) and bar plots showing the contributions of variables to dimension 1 (**b**) and dimension 2 (**c**) obtained from PCA analysis of the second evaluation screen of reaction conditions. The dotted red line in graphs (**b**,**c**) indicates the mean value.

The scree plot and the corresponding bar plots showing the contributions of variables (benzodiazepines) to dimensions 1 and 2, derived from PCA of the second evaluation screen of the reaction conditions, are presented in Figure 5.

The scree plot shows that the initial three principal components accounted for approximately 97% of the total variance in the *RRF* dataset for this second evaluation screen. The bar plot illustrating the variable contributions to dimension 1 revealed that AHM, OXA, and DES had the highest impact (around 14%) on observed variance, followed by NOR, TEM, and AHA, with contributions ranging from 11–12%. LOR exhibited a moderately distinct silylation response, contributing approximately 7% to the variability in dimension 1. In contrast, NIT, CLO, and BRO made minimal contributions (around 1%), indicating an insignificant impact on silylation responses, while being substantial contributors to observed variance in dimension 2 (around 27%), suggesting they played a more significant role in differentiating experimental conditions. The remaining benzodiazepines minimally contributed to the variance in dimension 2. The contribution analysis was not performed for benzodiazepine molecules toward dimension 3 because of their relatively lower contributions (10%) to the overall variance.

**Figure 6:**
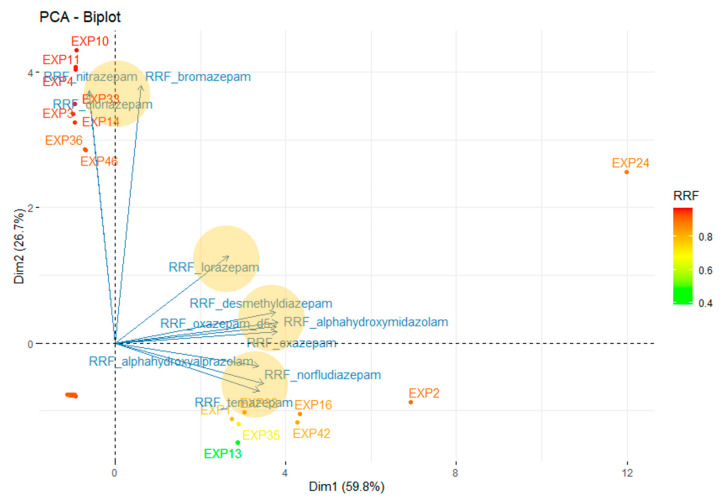
Scores biplot and distinct clusters derived from PCA of the second silylation panel for reaction conditions using 50 µL of BSTFA + 1% TMCS as the silylating agent. Clusters are indicated by orange circles.

The biplot for dimensions 1 and 2 in Figure 6 provides a clearer illustration of the cluster behavior derived from the PCA of the second dataset. In this biplot, the vectors exhibit a less radial distribution than in the previous dataset, indicating a more linear relationship between the variables and dimensions. Four distinct cluster regions can be distinguished.

NIT, CLO, and BRO form a cluster of eigenvectors in the upward direction, which aligns with experiments 3–4, 10–11, 14, 36, and 46. These experimental conditions significantly affected the silylation response in these benzodiazepines. This response can be attributed to the occurrence of comparable steric and electronic effects on the silylation reactions when BSTFA is used as the silylating reagent. These derivatives have only a low-acidic NH group for silylation, which may be the primary reason for their low activity against BSTFA. Notably, the eigenvectors of NIT and CLO overlapped, indicating almost identical silylation responses under these experimental conditions.

In contrast, the DES, OXA, and OXA-d5 eigenvectors form a distinct cluster that points almost perpendicular to the previous cluster. This result demonstrates that the silylation responses of these benzodiazepines are influenced by different experimental conditions than those of NIT, BRO, and CLO. In addition, TEM, NOR, and AHA aligned with experiments 2, 16, 32, and 42, forming another cluster. Finally, LOR does not align with any eigenvector cluster, which implies that its response may be unique to a specific set of experimental conditions not captured by the other clusters.

#### 2.2.3. ANOVA Boxplots and Dendrogram Analysis

**Figure 7:**
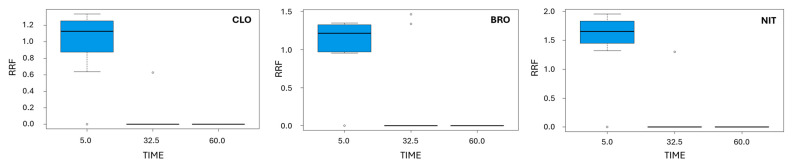
ANOVA boxplots for the silylation response of CLO, BRO, and NIT, indicating that reaction time has the most significant effect.

The ANOVA boxplot analysis revealed significant differences (*p* < 0.05) only for the clusters formed by NIT, CLO, and BRO, indicating that specific experimental conditions significantly influenced their silylation response. Figure 7 shows that reaction time had the most significant effect on the silylation response of NIT, CLO, and BRO. These derivatives exhibited higher *RRF* values and a wider distribution of data points at 5 min, indicating that shorter reaction durations have a more significant influence on silylation response. The effects of reaction temperature and catalyst amount on the silylation response of NIT, CLO, and BRO were relatively minor.

**Figure 8:**
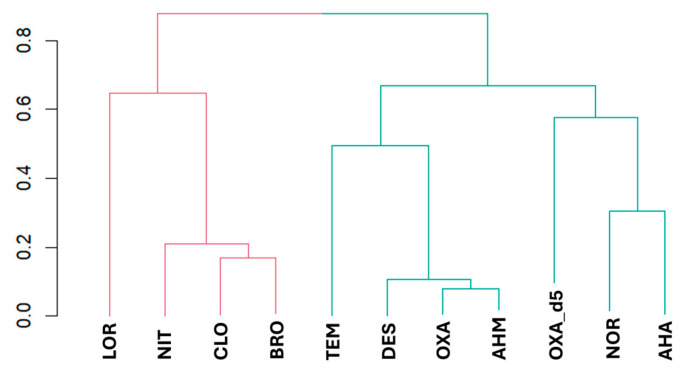
Hierarchical clustering analysis of benzodiazepines based on their silylation response (*RRF* values) under different experimental conditions. The length of the branches in the dendrogram represents the dissimilarity between clusters; shorter branches indicate greater similarity.

Hierarchical clustering analysis was performed to group the benzodiazepines according to their *RRF* values under different experimental conditions. The dendrogram in Figure 8 illustrates the hierarchical relationships among the benzodiazepines, showing two primary branches that indicate significant differences in the experimental conditions and silylation responses. One branch includes LOR, NIT, CLO, and BRO, while the other consists of TEM, DES, OXA, OXA-d5, AHI, NOR, and AHA. The primary branches were further divided into subclusters based on variations in the experimental conditions affecting the silylation response. Shorter branches indicate greater similarity between benzodiazepine clusters under different experimental conditions.

### 2.3. Determination of Benzodiazepines in Real Forensic Samples

An applied study was conducted to detect benzodiazepines in forensic samples using gas chromatography-mass spectrometry (GC-MS). The silylation reaction involved 50 μL of BSTFA + 1% TMCS, 50 μL of EA as the solvent, a reaction temperature of 80 °C, and a reaction time of 20 min without any catalyst or co-solvent. These conditions were selected based on a comparative analysis of the results of the two evaluation studies that yielded the highest benzodiazepine silylation coverage.

The study involved analyzing 30 postmortem urine samples from individuals suspected of having consumed benzodiazepines. Table 3 presents the results of the pharmacological analysis of these samples, along with basic information about the individuals (age, gender, and cause of death).

The application of the study to real cases allowed for the detection and identification of structurally diverse benzodiazepines in several of the postmortem urine samples tested, providing crucial information for forensic investigations. Notably, all 30 cases tested positive for benzodiazepines using preliminary EMI analysis, but the optimized silylation method led to the GC-MS confirmation of benzodiazepines in only half of the cases. These findings underscore the importance of accurate and sensitive analytical techniques, like derivatized-optimized GC-MS spectrometry, in determining benzodiazepines in forensic samples.

Benzodiazepines confirmed to be present in the postmortem urine samples included oxazepam (OXA), temazepam (TEM), norfludiazepam (NOR), desmethyldiazepam (DES), and alpha-hydroxyalprazolam (AHA). Furthermore, derivatives of benzodiazepines without active hydroxyl or amino groups, like diazepam (DIA), midazolam (MID), and etizolam (ETI), were also detected in the study. The identification of ETI was a particularly noteworthy finding because it is not a conventional benzodiazepine, yet it produces similar effects and is becoming more commonly used as a recreational alternative to benzodiazepines [22]. ETI is presently categorized as a designer benzodiazepine—a type of benzodiazepine analog synthesized to mimic the effects of traditional benzodiazepines [23].

The derivatization methodology using BSTFA + 1% TMCS as the silylating agent could silylate multiple compounds bearing hydroxyl or amino groups. Compounds identified in the study other than benzodiazepines that have active functional groups susceptible to silylation with BSTFA + 1% TMCS are marked with asterisks (*) in Table 3.

## 3. Materials and Methods

### 3.1. Reagents and Solvents

Benzodiazepine reference standards of clonazepam (315.72 g/mol, ≥98.5%, CAS No 1622-61-3), lorazepam (321.16 g/mol, ≥98.5%, CAS No 846-49-1), oxazepam (286.72 g/mol, ≥98.5%, CAS No 604-75-1), oxazepam-d5 (291.74 g/mol, ≥98.5%, CAS No 65854-78-6), bromazepam (316.16 g/mol, ≥98.5%, CAS No 1812-30-2), nitrazepam (281.27 g/mol, ≥98.5%, CAS No 146-22-5), temazepam (300.75 g/mol, ≥98.5%, CAS No 846-50-4), alpha-hydroxymidazolam (341.77 g/mol, ≥98.5%, CAS No 59468-90-5), norfludiazepam (288.71 g/mol, ≥98.5%, CAS No 2886-65-9), desmethyldiazepam (nordazepam) (270.75 g/mol, ≥98.5%, CAS No 1088-11-5), and alpha-hydroxyalprazolam (324.77 g/mol, ≥98.5%, CAS No 37115-43-8) were purchased from Lipomed (Arlesheim, Switzerland). HPLC-grade solvents such as methanol and dichloromethane were obtained from Mallinckrodt (Dublin, Ireland) and Scharlau (Barcelona, Spain), respectively. Acetonitrile (41.05 g/mol, ≥99.8%, CAS No 75-05-8), acetic acid (60.05 g/mol, ≥99.7%, CAS No 64-19-7), and isopropyl alcohol (60.10 g/mol, ≥99.0%, CAS No 67-63-0) were purchased from J.T. Baker^®^ (Radnor, PA, USA). Pyridine (79.1 g/mol, ≥99.5%, CAS No 110-86-1), ammonium hydroxide (28.0–30.0% as NH_3_ in water), and ethyl acetate (88.11 g/mol, ≥99.8%, CAS No 141-78-6) were sourced from Merck (Rahway, NJ, USA). An analytical standard of *N*,*O*-bis(trimethylsilyl)trifluoroacetamide (BSTFA) containing 1% TMCS (257.40 g/mol, CAS No 25561-30-2) was purchased from Supelco (Burlington, MA, USA).

Stock solutions of each benzodiazepine compound and diazepam (internal standard) at 1000 µg/mL were prepared in methanol and stored at −18 °C. Diazepam (284.75 g/mol, ≥98.5%, CAS No 439-14-5) was chosen as the internal standard (IS) because of its structural similarity with the analytes under study and lack of active groups for derivatization, which facilitates chromatographic response normalization. Working solutions containing 10 µg/mL of each target compound and IS were also prepared for testing derivatization procedures. Each replicate consisted of transferring 50 µL of the working standard solution along with 50 µL of IS solution (10 µg/mL) to a 1.5 mL reaction vial, followed by evaporation to dryness under a gentle stream of nitrogen before derivatization analysis using mass-coupled gas chromatography (GC-MS) under optimal conditions.

### 3.2. Silylation Derivatization and Benzodiazepine Detection by GC-MS

Derivatization reactions were performed using a Labconco RapidVap Vacuum Dry Evaporation System^®^ (Kansas City, MO, USA). Subsequent sample analyses were conducted using an Agilent 7890B gas chromatograph (Santa Clara, CA, USA) coupled to an Agilent 5975C mass (GC-MS) spectrometer. All GC-MS measurements were performed using an Rtx-5 fused silica capillary column (30 µm, 0.25 mm I.D., 0.25 µm film thickness, Restek, Mount Airy, PA, USA). The oven temperature program included initial heating at 80 °C for 1 min, followed by a gradual increase from 100 °C to 300 °C at a rate of 4 °C/min and sustained heating at 300 °C for 4 min. The carrier gas (helium) was maintained at a constant flow rate of 2.0 mL/min. The injector and detector were operated at 270 °C and 250 °C, respectively. Injections (1 µL) were performed in pulsed splitless injector mode while maintaining the transfer line at 300 °C. Mass spectrometric analysis was performed in full scan mode with electron impact ionization set to 70 eV over a mass range of 50–500 amu. The derivatization product mass spectra were recorded and matched against the NIST library using their retention times and characteristic MS spectra during derivatization optimization. Each derivatization was performed in triplicate and analyzed under the same chromatographic conditions.

### 3.3. Experimental Design for Derivatization Trials

To identify the optimal experimental conditions for the silylation of benzodiazepines using BSTFA + 1% TMCS, two stages were designed to evaluate the experimental variables (see Table 4). Each variable was tested with two extreme values and one in the middle. During the first evaluation stage, we assessed the impact of BSTFA + 1% TMCS, reaction temperature, reaction time, and volume of ethyl acetate as an anhydrous organic solvent. Table 4 presents the varied reaction conditions for this phase, resulting in 27 different sets of conditions (i.e., experiments). Each set was evaluated using eight benzodiazepines: lorazepam (LOR), oxazepam (OXA), bromazepam (BRO), temazepam (TEM), norfludiazepam (NOR), alpha-hydroxymidazolam (AHM), desmethyldiazepam (DES), and alpha-hydroxyalprazolam (AHA). The second stage further refined the experimental conditions for the silylation reaction by focusing on specific variables that showed promising results in the initial evaluation stage. In addition, variables such as the volume of acetonitrile and the addition of pyridine as a reaction catalyst were included. In this phase, five experimental conditions were evaluated (see Table 4): (i) the volume of ethyl acetate; (ii) the volume of acetonitrile combined with a fixed volume (50 µL) of BSTFA + 1% TMCS to improve reaction efficiency; (iii) the addition of pyridine as a catalyst, as it can activate the acidic hydrogens through an S_N_2 mechanism, thus shifting the equilibrium of the reaction toward the product side; (iv) reaction time; and (v) reaction temperature. These last two variables are critical because they can lead to the incomplete formation of mono-TMS and di-TMS derivatives. In total, 46 factor combinations (i.e., experiments) were tested on 10 different benzodiazepines in the second phase of the experimental design.

The effectiveness of the derivatization reaction was expressed as the relative response factor (*RRF*) [24,25], which was calculated using Equation (1).

(1) $$RRF=\frac{{RF}_{A}}{{RF}_{IS}}=\frac{\left(A_{A}/C_{A}\right)}{\left(A_{IS}/C_{IS}\right)}=\frac{\left(A_{A}\times C_{IS}\right)}{\left(A_{IS}\times C_{A}\right)},$$

where ${RF}_{A}$ and ${RF}_{IS}$ are response factors for the analyte (*A*) and internal standard (*IS*), respectively; $A_{A}$ is the target analyte base peak area, $A_{IS}$ is the internal standard base peak area (i.e., diazepam), $C_{A}$ is the target analyte concentration, and $C_{IS}$ is the internal standard concentration. During derivatization optimization, all determinations were performed using the same GC-MS conditions. The mean *RRFs* were estimated for *n* = 3 and RSD < 3%.

### 3.4. Extraction and Analysis of Forensic Samples

Postmortem urine samples from autopsies were obtained from the Toxicology Laboratory of the Institute of Legal Medicine and Forensic Sciences in Pereira, Colombia. Drug-free urine samples were collected from healthy subjects who were not using drugs. Both types of samples were stored in polypropylene bottles at −20 °C until analysis. The samples were previously analyzed by immunoassay using EMIT^®^ to screen for the presence of drugs of abuse and their metabolites, including benzodiazepines, cocaine, opioids, cannabinoids, amphetamines, methadone, ketamine, and barbiturates. Positive samples were then sent for confirmation. The hydrolysis process involved adding 300 μL of sodium acetate buffer (pH 4.5) to 2 mL of urine, followed by 50 μL of β-glucuronidase (from *Helix pomatia*) and incubation at 56 °C for 2 h. Finally, the pH was adjusted to 10 by adding 400 μL of sodium tetraborate buffer.

The extraction procedure involved activating the cartridge by sequentially passing 3 mL of methanol and 2 mL of distilled water through the column without allowing the adsorbent to dry. Subsequently, 3 mL of the pre-conditioned sample was loaded and washed with 3 mL of a mixture of water, methanol, and 0.01 M hydrochloric acid (6:3:1), which was then left to dry under vacuum for 15 min. Finally, a dichloromethane-isopropanol (9:1) elution solvent (3 mL) was added to the column and allowed to pass by gravity; thereafter, the eluate was collected and evaporated under a gentle stream of nitrogen for subsequent derivatization and GC-MS analysis. The applied validation methodology is described in more detail in Appendix A.

### 3.5. Statistical Analysis

As described above, the statistical analysis of the experimental data was conducted in two main stages (Table 4). Both experimental stages involved multivariate analysis of the individual and combined effects of the experimental variables on the relative response factor (*RRF*). These stages aimed to determine whether principal component analysis (PCA) and cluster analysis (CA) are useful tools for selecting the most efficient conditions for derivatizing benzodiazepines and their metabolites [26,27]. To achieve this, ANOVA, PCA, and cluster dendrograms were performed using Rstudio software (Version 4.1.2) [28]. All statistical tests were performed on the normalized *RRFs* (Equation (1)) calculated for all benzodiazepine derivatives.

## 4. Conclusions

This study successfully demonstrated the effectiveness of BSTFA + 1% TMCS as a silylating agent for the derivatization of benzodiazepines for GC-MS analysis in forensic applications. The multivariate optimization revealed that a higher BSTFA + 1% TMCS concentration, coupled with an appropriate volume of ethyl acetate, significantly enhances silylation efficiency. Notably, the addition of acetonitrile and a catalyst (pyridine) did not notably alter the silylation outcomes under the tested conditions.

The analytical approach incorporating principal component analysis (PCA) revealed diverse benzodiazepine response patterns, highlighting the need for tailored experimental conditions to achieve optimal derivatization. Hierarchical clustering analysis (HCA) further categorizes benzodiazepines based on their silylation responses, emphasizing the importance of individualized analysis parameters.

The practical application of the optimized conditions to forensic samples confirmed the proficiency of GC-MS in detecting and identifying a variety of benzodiazepine structures, including designer drugs like etizolam. This underscores the method’s potential to distinguish traditional benzodiazepines from emerging analogs, which is an advancement that is crucial for the evolving field of forensic toxicology. However, the high sensitivity of the silylation reaction with BSTFA + 1% TMCS to factors such as temperature, reaction time, and solvent quantity still requires careful control to ensure comprehensive derivatization and consistent quantification across the complete benzodiazepine spectrum.

The proposed methodology offers an economical and reliable alternative for the targeted and nontargeted analysis of a variety of forensically relevant substances, including benzodiazepines, opioids, cocaine, antidepressants, cannabinoids, ketamine, and amphetamine-type drugs, along with their metabolites in forensic matrices. Further studies should incorporate the use of high-resolution GC-MS (e.g., GC-MS/MS and GC-QTOF) to leverage the resolving power of high-resolution mass spectrometry in conjunction with the selectivity of the derivatization reaction.

In conclusion, this study not only enhances the understanding of benzodiazepine silylation dynamics but also provides a foundation for improving drug detection methodologies. Future research should investigate the silylation behavior of additional benzodiazepine analogs with BSTFA + 1% TMCS and explore the method’s adaptability to other forensic compounds. Likewise, further studies should evaluate the short- and long-term stability of TMS derivatives, particularly when samples need to be stored for extended periods.

## Figures and Tables

**Table 1:** Normalized *RRF* values under different experimental conditions for the silylation reaction of benzodiazepines.

Exp. N°	BSTFA + 1% TMCS [µL]	EA [µL]	Temp [°C]	Time [min]	Normalized Experimental *RRF* Values							
					LOR	OXA	BRO	TEM	NOR	AHM	DES	AHA
1	25	50	60	5.0	0.000	0.000	0.000	0.000	0.000	0.000	0.000	0.000
2	25	25	60	32.5	0.000	0.000	0.000	1.368	0.000	0.814	0.000	1.522
3	0	25	30	32.5	0.000	0.000	0.000	0.000	0.000	0.000	0.000	0.000
4	50	25	30	32.5	0.366	0.395	0.058	0.695	1.046	0.821	1.105	1.428
5	25	25	30	5.0	0.218	0.217	0.093	0.714	0.500	0.738	0.739	1.174
6	25	25	90	5.0	3.089	1.582	0.063	4.635	3.691	2.817	5.406	5.986
7	25	50	30	32.5	0.000	0.000	0.000	1.191	0.000	0.758	0.000	1.329
8	25	0	60	60.0	0.000	0.000	0.000	0.726	0.000	0.647	0.000	1.213
9	25	25	30	60.0	0.000	0.000	0.000	1.017	0.000	0.349	0.000	0.951
10	25	0	90	32.5	0.000	0.000	0.000	0.000	0.000	0.077	0.000	0.077
11	50	0	60	32.5	0.000	0.083	0.000	1.260	0.400	0.765	0.682	1.712
12	0	50	60	32.5	0.000	0.000	0.000	0.034	0.000	0.169	0.000	0.174
13	25	50	90	32.5	0.000	0.000	0.000	1.551	0.000	0.779	0.000	1.423
14	25	50	60	60.0	0.000	0.000	0.000	1.460	0.000	0.767	0.000	1.441
15	25	25	90	60.0	0.000	0.000	0.000	0.725	0.000	0.426	0.000	0.356
16	50	25	60	60.0	0.000	0.000	0.000	1.617	0.041	0.871	0.072	1.851
17	25	25	60	32.5	0.000	0.000	0.000	1.429	0.000	0.809	0.000	1.603
18	0	25	90	32.5	0.000	0.000	0.000	0.033	0.000	0.118	0.000	0.181
19	0	25	60	60.0	0.000	0.000	0.000	0.023	0.000	0.135	0.000	0.107
20	25	0	30	32.5	0.000	0.000	0.000	1.086	0.000	0.718	0.000	1.055
21	50	25	60	5.0	0.903	1.125	0.260	0.796	1.543	0.907	2.127	1.750
22	0	0	60	32.5	0.000	0.000	0.000	0.028	0.000	0.148	0.000	0.125
23	50	25	90	32.5	0.000	0.000	0.000	1.499	0.150	0.914	0.194	1.744
24	50	50	60	32.5	1.135	1.257	0.309	0.867	9.858	0.910	2.782	1.746
25	25	25	60	32.5	0.000	0.000	0.000	1.351	0.059	0.821	0.038	1.532
26	25	0	60	5.0	0.000	0.018	0.000	1.252	0.324	0.839	0.326	1.718
27	0	25	60	5.0	0.000	0.000	0.000	0.031	0.000	0.159	0.000	0.090

**Table 2:** Dataset of standardized *RRF* values obtained from the second evaluation of silylation conditions for benzodiazepines using BSTFA + 1% TMCS at a constant volume of 50 µL.

Exp	EA	ACN	Temp.	Time	Pyr	CLO	LOR	OXA	BRO	OXA-d5	NIT	TEM	AHM	NOR	DES	AHA
1	25	25	60	32.5	25	0.000	0.000	16.157	0.000	1.567	0.000	76.774	12.947	14.404	17.952	5.999
2	25	0	60	32.5	0	0.000	0.000	51.795	0.000	5.641	0.000	87.297	17.636	41.934	52.306	8.867
3	25	25	60	5.0	0	1.117	0.989	1.336	0.984	0.168	1.695	1.544	0.484	1.550	2.269	0.678
4	0	25	60	5.0	25	1.261	1.257	1.483	1.291	0.162	1.811	1.719	0.525	1.532	3.253	0.687
5	0	25	60	32.5	0	0.000	0.000	0.185	0.000	0.016	0.000	1.537	0.353	0.273	0.316	0.277
6	25	25	90	60.0	25	0.000	0.000	0.000	0.000	0.000	0.000	0.019	0.022	0.000	0.000	0.024
7	25	50	60	32.5	50	0.000	0.000	0.928	0.000	0.091	0.000	1.453	0.295	0.796	1.223	0.368
8	25	50	90	32.5	25	0.000	0.000	0.001	0.000	0.000	0.000	0.065	0.056	0.000	0.000	0.040
9	0	25	60	60.0	25	0.000	0.000	0.000	0.000	0.000	0.000	1.337	0.115	0.000	0.000	0.052
10	50	25	60	5.0	25	1.339	1.921	1.013	1.315	0.230	1.958	1.670	0.494	1.560	2.496	0.885
11	25	0	60	5.0	25	1.244	0.779	1.534	1.333	0.169	1.859	1.781	0.536	1.629	3.348	0.845
12	0	25	30	32.5	25	0.000	0.000	0.341	0.000	0.028	0.000	2.526	0.528	0.659	0.628	0.744
13	50	50	60	32.5	25	0.000	0.000	3.638	0.000	0.304	0.000	90.877	17.671	7.122	8.608	13.230
14	25	25	60	5.0	50	1.120	1.493	1.253	0.953	0.168	1.572	1.628	0.488	1.114	1.631	0.861
15	25	25	60	32.5	25	0.000	0.000	0.022	0.000	0.002	0.000	1.692	0.179	0.022	0.024	0.053
16	25	50	60	32.5	0	0.000	0.000	36.032	0.000	3.293	0.000	96.398	14.417	24.278	25.573	5.083
17	50	25	60	32.5	0	0.000	0.000	0.444	0.000	0.037	0.000	2.030	0.167	0.261	0.224	0.050
18	50	25	60	32.5	50	0.000	0.000	0.000	0.000	0.000	0.000	0.016	0.014	0.000	0.000	0.008
19	25	50	30	32.5	25	0.000	0.000	0.000	0.000	0.000	0.000	0.000	0.000	0.000	0.000	0.000
20	25	25	30	32.5	50	0.000	0.000	0.000	0.000	0.000	0.000	1.345	0.335	0.000	0.000	0.359
21	25	25	30	32.5	0	0.000	0.000	0.000	0.000	0.000	0.000	0.039	0.004	0.000	0.000	0.000
22	25	25	30	5.0	25	0.000	0.000	0.000	0.000	0.000	0.000	0.918	0.277	0.000	0.000	0.332
23	25	25	90	32.5	50	0.000	0.000	0.000	0.000	0.000	0.000	0.000	0.000	0.000	0.000	0.000
24	0	50	60	32.5	25	0.000	32.092	71.475	1.464	7.410	0.000	82.869	16.212	78.669	104.439	9.722
25	25	0	60	60.0	25	0.000	0.000	0.000	0.000	0.000	0.000	1.307	0.112	0.000	0.000	0.045
26	25	25	60	60.0	0	0.000	0.000	0.118	0.000	0.009	0.000	1.602	0.181	0.090	0.067	0.078
27	25	50	60	60.0	25	0.000	0.000	0.001	0.000	0.000	0.000	1.405	0.182	0.000	0.000	0.090
28	25	25	60	60.0	50	0.000	0.000	0.000	0.000	0.000	0.000	1.206	0.193	0.000	0.000	0.068
29	25	25	30	60.0	25	0.000	0.000	0.000	0.000	0.000	0.000	0.000	0.000	0.000	0.000	0.000
30	0	25	60	32.5	50	0.000	0.000	0.277	0.000	0.023	0.000	1.746	0.215	0.290	0.333	0.073
31	25	25	90	32.5	0	0.000	0.000	0.000	0.000	0.000	0.000	1.124	0.263	0.000	0.000	0.294
32	0	0	60	32.5	25	0.000	0.000	20.343	0.000	1.794	0.000	82.401	10.372	22.033	22.626	5.317
33	25	50	60	5.0	25	1.138	1.422	1.392	1.137	0.162	1.615	1.605	0.457	1.432	2.171	0.653
34	0	25	90	32.5	25	0.000	0.000	0.172	0.000	0.019	0.000	1.694	0.299	0.303	0.323	0.460
35	25	0	60	32.5	50	0.000	0.000	15.738	0.000	1.481	0.000	95.628	13.957	15.586	15.176	5.643
36	25	25	90	5.0	25	0.638	1.367	2.849	1.347	0.287	1.323	1.445	0.331	2.403	3.525	0.546
37	50	0	60	32.5	25	0.000	0.000	0.499	0.000	0.047	0.000	1.517	0.352	0.493	0.699	0.218
38	50	25	90	32.5	25	0.000	0.000	0.461	0.000	0.039	0.000	1.623	0.331	0.425	0.528	0.439
39	25	25	60	32.5	25	0.000	0.000	0.012	0.000	0.001	0.000	1.619	0.128	0.011	0.003	0.061
40	50	25	30	32.5	25	0.000	0.000	0.001	0.000	0.000	0.000	0.000	0.000	0.000	0.000	0.000
41	25	25	60	32.5	25	0.000	0.000	0.001	0.000	0.000	0.000	1.422	0.104	0.000	0.000	0.043
42	25	25	60	32.5	25	0.000	0.000	24.770	0.000	2.293	0.000	82.648	17.779	23.682	25.286	9.424
43	25	0	90	32.5	25	0.000	0.000	0.055	0.000	0.005	0.000	1.676	0.350	0.118	0.123	0.449
44	25	25	60	32.5	25	0.000	0.000	0.022	0.000	0.002	0.000	1.830	0.113	0.020	0.021	0.042
45	50	25	60	60.0	25	0.000	0.000	0.019	0.000	0.002	0.000	1.744	0.068	0.024	0.034	0.065
46	25	0	30	32.5	25	0.626	2.204	2.468	1.337	0.282	1.302	1.346	0.332	2.168	2.925	0.534

**Table 3:** Results of the pharmacological analysis of 30 postmortem urine samples (with initial suspected presence of benzodiazepines) using the optimized silylating method with BSTFA + 1% TMCS.

Case	Gender ^1^	Age	Cause of Death ^2^	Benzodiazepine Detected ^3^	Other Substances Detected
1	M	67	TA	-	Ketamine, Norketamine *
2	F	55	UD	-	Caffeine, Ketamine
3	F	52	S	-	Ketamine
4	M	51	UD	-	Caffeine, Benzoylecgonine *, Ecgonine methyl ester *
5	M	14	F	DIA, TEM *	Benzoylecgonine *, 11-Nor-9-carboxy-Δ9-THC *
6	M	18	F	DIA	Cocaine, Benzoylecgonine *, Ecgonine methyl ester *
7	M	47	S	-	Clozapine *, Methadone, Zolpidem
8	M	63	UD	-	Methadone, Morphine *
9	M	61	F	-	Methadone, Cocaine, Caffeine
10	F	26	SA/F	NOR *	Caffeine, Ketamine, Norketamine *
11	M	23	S	-	Clozapine *, Amitriptyline, Tramadol *
12	M	49	F	ETI	-
13	M	24	TA	-	Amitriptyline, Nortriptyline *
14	M	59	F	-	Ecgonine methyl ester *, Ketamine, Methylenedioxymethamphetamine *
15	F	15	S	NOR *, OXA *, TEM *	-
16	F	38	SA/F		Methotrimeprazine, 11-Nor-9-carboxy-Δ9-THC *
17	M	16	F		Amitriptyline
18	M	21	F	MID	-
19	M	16	TA	DES *	-
20	M	31	S	AHA *	-
21	M	45	TA	-	Clozapine *
22	M	60	TA	AHM *, MID	Ketamine
23	M	20	F	AHM *, MID	Benzoylecgonine *
24	M	56	UD	NOR *	Benzoylecgonine *, Ecgonine methyl ester *
25	M	30	TA	AHM *	Ketamine, Norketamine *, Fentanyl
26	M	29	SFI	NOR *	Ketamine, Norketamine, Tenanfetamine *
27	M	63	TA	MID	11-Nor-9-carboxy-Δ9-THC *
28	F	56	UD	-	Codeine, Tramadol *, Methotrimeprazine
29	M	59	TA	-	Tramadol, Methotrimeprazine, Methadone,
30	M	30	TA	AHM *, MID	Fentanyl, Lidocaine

^1^ Sex-based classification: M = Male, F = Female. ^2^ TA = Traffic accident, S = Suicide, F = Firearm, SA/F = Sexual assault/feminicide, SFI = Sharp force injury, and UD = Undetermined, no apparent cause of death. ^3^ DIA = Diazepam, TEM = Temazepam, NOR = Norfludiazepam, ETI = Etizolam, OXA = Oxazepam, MID = Midazolam, DES = Desmethyldiazepam, AHA = Alpha-hydroxyalprazolam, and AHM = Alpha-hydroxymidazolam. * Compound with active group(s) for derivatization with BSTFA + 1% TMCS.

**Table 4:** Experimental conditions for evaluating the silylation reaction variables in two stages.

Experiment Stage	Reaction Time (min)	Reaction Temperature (°C)	BSTFA + 1% TMCS Volume (µL)	Ethyl Acetate Volume (µL)	ACN Volume (µL)	Pyridine Volume (µL)
Stage 1	5, 32.5 and 60	30, 60 and 90	0, 25 and 50	0, 25 and 50	NA *	NA *
Stage 2	5, 32.5 and 60	30, 60 and 90	fixed at 50	0, 25 and 50	0, 25 and 50	0, 25 and 50

* Not applicable.

## Data Availability

Data will be made available on request.

## Supplementary Materials

The following supporting information can be downloaded at https://www.mdpi.com/article/10.3390/molecules29245884/s1, ANOVA analysis (screening). Figure S1: Response surface graphs effects on *RRF* of clonazepam. Reaction time has statistical significance; Figure S2: Response surface graphs effects on *RRF* of lorazepam. Interaction between acetonitrile and ethyl acetate volumes had statistical significance; Figure S3: Response surface graphs effects on *RRF* of bromazepam. Reaction time has statistical significance; Figure S4: Response surface graphs effects on *RRF* of alpha-hydroxymidazolam. No significant effect on *RRF*; Figure S5: Response surface graphs effects on *RRF* of nitrazepam. Reaction time has statistical significance.

## Appendix A. Validation Methodology

- **Selectivity**

To assess selectivity, blank urine samples were analyzed to ensure the absence of interfering peaks or contaminants that could impact the detection and quantification of benzodiazepines. The benzodiazepine was evaluated, and potential interferents were added to a blank urine sample at a final concentration of 10 mg/L each. Substances commonly associated with benzodiazepine use, such as cocaine, benzoylecgonine, morphine, 6-monoacetylmorphine, imipramine, chlorpromazine, cocaethylene, codeine, cannabidiol, and 11-nor-9-carboxy-delta-9-tetrahydrocannabinol, were chosen as interfering agents. Ten aliquots were then extracted using the previously described methodology and analyzed by GC-MS in full scan mode. The method was deemed selective because it could extract and identify analytes of interest in the presence of potential interferents and endogenous compounds.

- **Linearity**

The linearity of the method was assessed by creating a range of standard solutions of benzodiazepines at various concentrations within the expected range. A calibration curve was then created using urine samples spiked with certified reference standards and the internal standard (oxazepam-d5, 20 mg/L) over concentration levels from 0.5 to 100 mg/L. Each concentration level was analyzed in triplicate, and regression parameters were calculated using the least squares method by measuring and plotting the ratio of analyte/internal standard responses on the ordinate against the concentration ratio on the abscissa.

- **Limits of Detection and Quantification**

The limit of detection (LoD) and limit of quantification (LoQ) were determined using the t99SLLMV methodology, originally described by the US EPA for water analysis [20]. This method offers up to 99% confidence in its two stages, considering instrumental noise (S/N), sensitivity to variability, extraction efficiency, matrix effects, and interferences.

- **Recovery**

Recovery experiments were carried out to evaluate the accuracy and efficiency of the silylation reaction for benzodiazepines. Urine samples with a concentration corresponding to the calculated limit of quantification for each analyte were prepared, and then each sample was analyzed in ten replicates using the previously optimized method. Another set comprised ten replicate samples injected into the chromatographic system without extraction. The absolute recovery was determined by calculating the mean ratio of extracted and non-extracted samples, where a response not extracted theoretically represented 100% recovery, indicating the accuracy and efficiency of the silylation reaction for benzodiazepines.
