# Supplementary material for: Insights into the Silylation of Benzodiazepines Using N,O-Bis(trimethylsilyl)trifluoroacetamide (BSTFA): In Search of Optimal Conditions for Forensic Analysis by GC-MS
Source: Molecules. 2024 Dec 13;29(24):5884. doi: 10.3390/molecules29245884 (PMC11678304; doi:10.3390/molecules29245884)
Supplement: Supplementary file 1 [file molecules-29-05884-s001.zip › molecules-3348430-supplementary.pdf]

Supporting Information

Insights into the silylation of benzodiazepines using N,O-bis(trimethylsilyl)trifluoroacetamide (BSTFA): in search of optimal conditions for forensic analysis by GC-MS

Eleazar Vargas Mena [1,*], Eliana R. Herrera Giraldo [2] and Jovanny A. Gómez Castaño [2,*]

[1] Grupo Ciencias Forenses, Instituto Nacional de Medicina Legal y Ciencias Forenses, Laboratorio de Toxicología Regional de Occidente, Pereira 660004, Avenida de las Américas No 98-25; eleazar.vargas@medicinalegal.gov.co

[2] Grupo Química-Física Molecular y Modelamiento Computacional (QUIMOL), Escuela de Ciencias Químicas, Universidad Pedagógica y Tecnológica de Colombia, Sede Tunja, Avenida Central del Norte, 150003, Boyacá; grupo.quimol@uptc.edu.co

* Correspondence: eleazar.vargas@medicinalegal.gov.co (E.V.M.); jovanny.gomez@uptc.edu.co (J.A.G.C.)

A. CHEMOMETRIC ANALYSIS (SCREENING) PART I

I. ANOVA analysis

ANOVA RRF of lorazepam

	Df	Sum Sq	Mean Sq	F Value	Pr(>F)	
BSTFA_VOL	1	0.482	0.4818	1.425	0.2500	
Temp	1	0.523	0.5231	1.547	0.2315	
Time	1	1.477	1.4768	4.367	0.0530	.
Ethyl_acet_vol	1	0.107	0.1074	0.318	0.5808	
BSTFA_VOL:Temp	1	0.033	0.0335	0.099	0.7570	
BSTFA_VOL:Time	1	0.204	0.2039	0.603	0.4488	
Temp:Time	1	2.061	2.0613	6.096	0.0252	*
BSTFA_VOL:Ethyl_acet_vol	1	0.322	0.3223	0.953	0.3434	
Temp:Ethyl_acet_vol	1	0.000	0.0000	0.000	1.0000	
Time:Ethyl_acet_vol	1	0.000	0.0000	0.000	1.0000	
Residuals	16	5.410	0.3381			

ANOVA RRF of oxazepam

	Df	Sum Sq	Mean Sq	F value	Pr(>F)	
BSTFA_VOL	1	0.6821	0.6821	5.484	0.0325	*
Temp	1	0.0783	0.0783	0.629	0.4392	
Time	1	0.7216	0.7216	5.801	0.0284	*
Ethyl_acet_vol	1	0.1114	0.1114	0.896	0.3579	
BSTFA_VOL:Temp	1	0.0390	0.0390	0.314	0.5831	
BSTFA_VOL:Time	1	0.3167	0.3167	2.546	0.1301	
Temp:Time	1	0.4654	0.4654	3.741	0.0710	.
BSTFA_VOL:Ethyl_acet_vol	1	0.3449	0.3449	2.773	0.1153	
Temp:Ethyl_acet_vol	1	0.0000	0.0000	0.000	1.0000	
Time:Ethyl_acet_vol	1	0.0001	0.0001	0.001	0.9798	
Residuals	16	1.9902	0.1244			

ANOVA RRF of bromazepam

	Df	Sum Sq	Mean Sq	F value	Pr(>F)	
BSTFA_VOL	1	0.03271	0.03271	8.936	0.00867	**
Temp	1	0.00065	0.00065	0.177	0.67950	
Time	1	0.01444	0.01444	3.946	0.06439	.
Ethyl_acet_vol	1	0.00794	0.00794	2.168	0.16032	
BSTFA_VOL:Temp	1	0.00085	0.00085	0.232	0.63645	
BSTFA_VOL:Time	1	0.01685	0.01685	4.604	0.04759	*
Temp:Time	1	0.00022	0.00022	0.061	0.80804	
BSTFA_VOL:Ethyl_acet_vol	1	0.02381	0.02381	6.504	0.02139	*
Temp:Ethyl_acet_vol	1	0.00000	0.00000	0.000	1.00000	
Time:Ethyl_acet_vol	1	0.00000	0.00000	0.000	1.00000	
Residuals	16	0.05857	0.00366			

ANOVA RRF of temazepam

	Df	Sum Sq	Mean Sq	F value	Pr(>F)	
BSTFA_VOL	1	3.613	3.613	5.005	0.0399	*
Temp	1	1.166	1.166	1.615	0.2220	
Time	1	0.288	0.288	0.400	0.5363	
Ethyl_acet_vol	1	0.047	0.047	0.065	0.8021	
BSTFA_VOL:Temp	1	0.149	0.149	0.206	0.6559	
BSTFA_VOL:Time	1	0.172	0.172	0.238	0.6325	
Temp:Time	1	4.437	4.437	6.146	0.0247	*
BSTFA_VOL:Ethyl_acet_vol	1	0.040	0.040	0.056	0.8165	
Temp:Ethyl_acet_vol	1	0.523	0.523	0.724	0.4073	
Time:Ethyl_acet_vol	1	0.986	0.986	1.366	0.2597	
Residuals	16	11.549	0.722			

Signif. codes: 0 ‘***’ 0.001 ‘**’ 0.01 ‘*’ 0.05 ‘.’ 0.1 ‘ ’ 1

ANOVA RRF of norfludiazepam

	Df	Sum Sq	Mean Sq	F value	Pr(>F)	
BSTFA_VOL	1	14.17	14.167	4.273	0.0553	.
Temp	1	0.44	0.439	0.132	0.7209	
Time	1	3.02	3.017	0.910	0.3543	
Ethyl_acet_vol	1	6.95	6.951	2.096	0.1670	
BSTFA_VOL:Temp	1	0.20	0.201	0.061	0.8087	
BSTFA_VOL:Time	1	0.56	0.564	0.170	0.6855	
Temp:Time	1	2.54	2.545	0.767	0.3940	
BSTFA_VOL:Ethyl_acet_vol	1	22.36	22.360	6.743	0.0195	*
Temp:Ethyl_acet_vol	1	0.00	0.000	0.000	1.0000	
Time:Ethyl_acet_vol	1	0.03	0.026	0.008	0.9302	
Residuals	16	53.05	3.316			

ANOVA RRF of alphahydroxymidazolam

	Df	Sum Sq	Mean Sq	F value	Pr(>F)	
BSTFA_VOL	1	1.656	1.6561	6.526	0.0212	*
Temp	1	0.255	0.2545	1.003	0.3315	
Time	1	0.428	0.4277	1.685	0.2126	
Ethyl_acet_vol	1	0.003	0.0030	0.012	0.9145	
BSTFA_VOL:Temp	1	0.000	0.0002	0.001	0.9803	
BSTFA_VOL:Time	1	0.000	0.0000	0.000	0.9908	
Temp:Time	1	1.001	1.0014	3.946	0.0644	.
BSTFA_VOL:Ethyl_acet_vol	1	0.004	0.0038	0.015	0.9037	
Temp:Ethyl_acet_vol	1	0.109	0.1094	0.431	0.5208	
Time:Ethyl_acet_vol	1	0.230	0.2300	0.906	0.3552	
Residuals	16	4.060	0.2537			

ANOVA RRF of desmethyldiazepam

	Df	Sum Sq	Mean Sq	F value	Pr(>F)	
BSTFA_VOL	1	4.040	4.040	3.636	0.0747	.
Temp	1	1.175	1.175	1.057	0.3191	
Time	1	6.058	6.058	5.452	0.0329	*
Ethyl_acet_vol	1	0.262	0.262	0.236	0.6337	
BSTFA_VOL:Temp	1	0.208	0.208	0.187	0.6712	
BSTFA_VOL:Time	1	1.056	1.056	0.950	0.3441	
Temp:Time	1	5.443	5.443	4.899	0.0417	*
BSTFA_VOL:Ethyl_acet_vol	1	1.102	1.102	0.992	0.3341	
Temp:Ethyl_acet_vol	1	0.000	0.000	0.000	1.0000	
Time:Ethyl_acet_vol	1	0.027	0.027	0.024	0.8791	
Residuals	16	17.777	1.111			

ANOVA RRF of alphahydroxyalprazolam

	Df	Sum Sq	Mean Sq	F value	Pr(>F)	
BSTFA_VOL	1	7.608	7.608	7.326	0.0156	*
Temp	1	1.222	1.222	1.176	0.2942	
Time	1	1.917	1.917	1.846	0.1931	
Ethyl_acet_vol	1	0.004	0.004	0.004	0.9526	
BSTFA_VOL:Temp	1	0.005	0.005	0.004	0.9479	
BSTFA_VOL:Time	1	0.002	0.002	0.002	0.9678	
Temp:Time	1	7.310	7.310	7.039	0.0174	*
BSTFA_VOL:Ethyl_acet_vol	1	0.000	0.000	0.000	0.9940	
Temp:Ethyl_acet_vol	1	0.287	0.287	0.276	0.6066	
Time:Ethyl_acet_vol	1	0.946	0.946	0.911	0.3540	
Residuals	16	16.616	1.039			

B. CHEMOMETRIC ANALYSIS PART II

I. ANOVA analysis

ANOVA RRF of clonazepam

	Df	Sum Sq	Mean Sq	F value	Pr(>F)	
Time	1	3.859	3.859	29.936	6.16e-06	***
Temp	1	0.000	0.000	0.000	0.994	
Pyridine_vol	1	0.000	0.000	0.000	0.998	
Ethyl_acet_vol	1	0.000	0.000	0.003	0.957	
ACN_vol	1	0.034	0.034	0.260	0.614	
Time:Temp	1	0.102	0.102	0.789	0.381	
Time:Pyridine_vol	1	0.000	0.000	0.000	0.997	
Temp:Pyridine_vol	1	0.000	0.000	0.000	1.000	
Time:Ethyl_acet_vol	1	0.002	0.002	0.012	0.914	
Temp:Ethyl_acet_vol	1	0.000	0.000	0.000	1.000	
Pyridine_vol:Ethyl_acet_vol	1	0.000	0.000	0.000	1.000	
Time:ACN_vol	1	0.003	0.003	0.022	0.883	
Temp:ACN_vol	1	0.098	0.098	0.761	0.390	
Pyridine_vol:ACN_vol	1	0.000	0.000	0.000	1.000	
Ethyl_acet_vol:ACN_vol	1	0.000	0.000	0.000	1.000	
Residuals	30	3.867	0.129			

ANOVA RRF of lorazepam

	Df	Sum Sq	Mean Sq	F value	Pr(>F)	
Time	1	5.3	5.32	0.257	0.61599	
Temp	1	0.0	0.04	0.002	0.96362	
Pyridine_vol	1	0.0	0.02	0.001	0.97811	
Ethyl_acet_vol	1	61.7	61.73	2.978	0.09467	.
ACN_vol	1	58.3	58.25	2.811	0.10402	
Time:Temp	1	0.5	0.47	0.023	0.88168	
Time:Pyridine_vol	1	0.1	0.06	0.003	0.95625	
Temp:Pyridine_vol	1	0.0	0.00	0.000	1.00000	
Time:Ethyl_acet_vol	1	0.1	0.11	0.005	0.94227	
Temp:Ethyl_acet_vol	1	0.0	0.00	0.000	1.00000	
Pyridine_vol:Ethyl_acet_vol	1	0.0	0.00	0.000	1.00000	
Time:ACN_vol	1	0.1	0.10	0.005	0.94421	
Temp:ACN_vol	1	1.2	1.21	0.059	0.81035	
Pyridine_vol:ACN_vol	1	0.0	0.00	0.000	1.00000	
Ethyl_acet_vol:ACN_vol	1	257.5	257.47	12.423	0.00138	**
Residuals	30	621.7	20.72			

ANOVA RRF of oxazepam

	Df	Sum Sq	Mean Sq	F value	Pr(>F)
Time	1	7	7.2	0.028	0.869
Temp	1	0	0.0	0.000	0.991
Pyridine_vol	1	321	321.4	1.235	0.275
Ethyl_acet_vol	1	486	486.2	1.868	0.182
ACN_vol	1	28	27.7	0.106	0.747
Time:Temp	1	2	2.0	0.008	0.930
Time:Pyridine_vol	1	0	0.0	0.000	0.999
Temp:Pyridine_vol	1	0	0.0	0.000	1.000
Time:Ethyl_acet_vol	1	0	0.1	0.000	0.988
Temp:Ethyl_acet_vol	1	0	0.1	0.000	0.985
Pyridine_vol:Ethyl_acet_vol	1	0	0.1	0.000	0.987
Time:ACN_vol	1	0	0.0	0.000	0.996
Temp:ACN_vol	1	1	1.5	0.006	0.941
Pyridine_vol:ACN_vol	1	0	0.2	0.001	0.977
Ethyl_acet_vol:ACN_vol	1	576	575.8	2.212	0.147
Residuals	30	7810	260.3		

ANOVA RRF of bromazepam

	Df	Sum Sq	Mean Sq	F value	Pr(>F)	
Time	1	4.367	4.367	24.108	3e-05	***
Temp	1	0.000	0.000	0.000	0.9954	
Pyridine_vol	1	0.000	0.000	0.000	0.9858	
Ethyl_acet_vol	1	0.130	0.130	0.715	0.4045	
ACN_vol	1	0.000	0.000	0.002	0.9682	
Time:Temp	1	0.453	0.453	2.503	0.1241	
Time:Pyridine_vol	1	0.000	0.000	0.001	0.9715	
Temp:Pyridine_vol	1	0.000	0.000	0.000	1.0000	
Time:Ethyl_acet_vol	1	0.000	0.000	0.001	0.9769	
Temp:Ethyl_acet_vol	1	0.000	0.000	0.000	1.0000	
Pyridine_vol:Ethyl_acet_vol	1	0.000	0.000	0.000	1.0000	
Time:ACN_vol	1	0.010	0.010	0.053	0.8195	
Temp:ACN_vol	1	0.447	0.447	2.466	0.1268	
Pyridine_vol:ACN_vol	1	0.000	0.000	0.000	1.0000	
Ethyl_acet_vol:ACN_vol	1	0.536	0.536	2.959	0.0957	.
Residuals	30	5.435	0.181			

ANOVA RRF of oxazepam-d5

	Df	Sum Sq	Mean Sq	F value	Pr(>F)	
Time	1	4.367	4.367	24.108	3e-05	***
Temp	1	0.000	0.000	0.000	0.9954	
Pyridine_vol	1	0.000	0.000	0.000	0.9858	
Ethyl_acet_vol	1	0.130	0.130	0.715	0.4045	
ACN_vol	1	0.000	0.000	0.002	0.9682	
Time:Temp	1	0.453	0.453	2.503	0.1241	
Time:Pyridine_vol	1	0.000	0.000	0.001	0.9715	
Temp:Pyridine_vol	1	0.000	0.000	0.000	1.0000	
Time:Ethyl_acet_vol	1	0.000	0.000	0.001	0.9769	
Temp:Ethyl_acet_vol	1	0.000	0.000	0.000	1.0000	
Pyridine_vol:Ethyl_acet_vol	1	0.000	0.000	0.000	1.0000	
Time:ACN_vol	1	0.010	0.010	0.053	0.8195	
Temp:ACN_vol	1	0.447	0.447	2.466	0.1268	
Pyridine_vol:ACN_vol	1	0.000	0.000	0.000	1.0000	
Ethyl_acet_vol:ACN_vol	1	0.536	0.536	2.959	0.0957	.
Residuals	30	5.435	0.181			

ANOVA RRF of nitrazepam

	Df	Sum Sq	Mean Sq	F value	Pr(>F)	
Time	1	8.750	8.750	31.149	4.52e-06	***
Temp	1	0.000	0.000	0.000	0.992	
Pyridine_vol	1	0.001	0.001	0.003	0.954	
Ethyl_acet_vol	1	0.001	0.001	0.005	0.945	
ACN_vol	1	0.149	0.149	0.532	0.472	
Time:Temp	1	0.437	0.437	1.557	0.222	
Time:Pyridine_vol	1	0.004	0.004	0.013	0.909	
Temp:Pyridine_vol	1	0.000	0.000	0.000	1.000	
Time:Ethyl_acet_vol	1	0.005	0.005	0.019	0.890	
Temp:Ethyl_acet_vol	1	0.000	0.000	0.000	1.000	
Pyridine_vol:Ethyl_acet_vol	1	0.000	0.000	0.000	1.000	
Time:ACN_vol	1	0.015	0.015	0.053	0.820	
Temp:ACN_vol	1	0.424	0.424	1.508	0.229	
Pyridine_vol:ACN_vol	1	0.000	0.000	0.000	1.000	
Ethyl_acet_vol:ACN_vol	1	0.000	0.000	0.000	1.000	
Residuals	30	8.427	0.281			

ANOVA RRF of temazepam

	Df	Sum Sq	Mean Sq	F value	Pr(>F)
Time	1	1	0.9	0.001	0.981
Temp	1	0	0.1	0.000	0.992
Pyridine_vol	1	490	490.1	0.340	0.564
Ethyl_acet_vol	1	364	364.4	0.252	0.619
ACN_vol	1	0	0.2	0.000	0.991
Time:Temp	1	0	0.1	0.000	0.995
Time:Pyridine_vol	1	0	0.1	0.000	0.995
Temp:Pyridine_vol	1	1	1.5	0.001	0.975
Time:Ethyl_acet_vol	1	0	0.1	0.000	0.995
Temp:Ethyl_acet_vol	1	2	1.5	0.001	0.974
Pyridine_vol:Ethyl_acet_vol	1	1	1.2	0.001	0.977

Time:ACN_vol	1	0	0.0	0.000	0.997
Temp:ACN_vol	1	0	0.0	0.000	0.997
Pyridine_vol:ACN_vol	1	2666	2666.5	1.847	0.184
Ethyl_acet_vol:ACN_vol	1	1975	1975.4	1.369	0.251
Residuals	30	43300	1443.3		

ANOVA RRF of alphahydroxymidazolam

	Df	Sum Sq	Mean Sq	F value	Pr(>F)
Time	1	0.5	0.46	0.010	0.922
Temp	1	0.0	0.00	0.000	0.995
Pyridine_vol	1	20.3	20.27	0.426	0.519
Ethyl_acet_vol	1	5.7	5.67	0.119	0.732
ACN_vol	1	2.0	1.99	0.042	0.839
Time:Temp	1	0.0	0.00	0.000	0.998
Time:Pyridine_vol	1	0.0	0.00	0.000	1.000
Temp:Pyridine_vol	1	0.1	0.09	0.002	0.966
Time:Ethyl_acet_vol	1	0.0	0.00	0.000	0.999
Temp:Ethyl_acet_vol	1	0.1	0.08	0.002	0.968
Pyridine_vol:Ethyl_acet_vol	1	0.0	0.00	0.000	0.999
Time:ACN_vol	1	0.0	0.01	0.000	0.992
Temp:ACN_vol	1	0.0	0.00	0.000	0.998
Pyridine_vol:ACN_vol	1	27.3	27.26	0.573	0.455
Ethyl_acet_vol:ACN_vol	1	32.9	32.94	0.692	0.412
Residuals	30	1427.8	47.59		

ANOVA RRF of norfludiazepam

	Df	Sum Sq	Mean Sq	F value	Pr(>F)
Time	1	8	7.7	0.031	0.861
Temp	1	0	0.0	0.000	0.995
Pyridine_vol	1	160	160.0	0.648	0.427
Ethyl_acet_vol	1	551	550.8	2.231	0.146
ACN_vol	1	50	50.2	0.203	0.655
Time:Temp	1	1	1.4	0.006	0.940
Time:Pyridine_vol	1	0	0.0	0.000	0.991
Temp:Pyridine_vol	1	0	0.0	0.000	1.000
Time:Ethyl_acet_vol	1	0	0.0	0.000	1.000
Temp:Ethyl_acet_vol	1	0	0.2	0.001	0.980
Pyridine_vol:Ethyl_acet_vol	1	0	0.0	0.000	0.993
Time:ACN_vol	1	0	0.0	0.000	0.995
Temp:ACN_vol	1	1	1.1	0.004	0.948
Pyridine_vol:ACN_vol	1	2	2.1	0.008	0.928
Ethyl_acet_vol:ACN_vol	1	625	625.2	2.532	0.122
Residuals	30	7407	246.9		

ANOVA RRF of desmethyldiazepam

	Df	Sum Sq	Mean Sq	F value	Pr(>F)	
Time	1	22	21.6	0.056	0.814	
Temp	1	0	0.1	0.000	0.990	
Pyridine_vol	1	243	243.3	0.634	0.432	
Ethyl_acet_vol	1	890	890.0	2.319	0.138	
ACN_vol	1	125	125.5	0.327	0.572	
Time:Temp	1	3	3.1	0.008	0.929	
Time:Pyridine_vol	1	0	0.1	0.000	0.988	
Temp:Pyridine_vol	1	0	0.0	0.000	1.000	
Time:Ethyl_acet_vol	1	0	0.2	0.000	0.984	
Temp:Ethyl_acet_vol	1	0	0.2	0.000	0.983	
Pyridine_vol:Ethyl_acet_vol	1	0	0.0	0.000	0.995	
Time:ACN_vol	1	0	0.3	0.001	0.976	
Temp:ACN_vol	1	2	2.0	0.005	0.943	
Pyridine_vol:ACN_vol	1	41	40.8	0.106	0.747	
Ethyl_acet_vol:ACN_vol	1	1365	1365.5	3.557	0.069	.
Residuals	30	11515	383.8			

ANOVA RRF of alpha-hydroxy alprazolam

	Df	Sum Sq	Mean Sq	F value	Pr(>F)
Time	1	1.6	1.602	0.115	0.737
Temp	1	0.0	0.005	0.000	0.985
Pyridine_vol	1	3.9	3.946	0.283	0.599
Ethyl_acet_vol	1	0.4	0.371	0.027	0.871
ACN_vol	1	3.3	3.301	0.237	0.630

Time:Temp	1	0.0	0.009	0.001	0.980
Time:Pyridine_vol	1	0.0	0.009	0.001	0.980
Temp:Pyridine_vol	1	0.1	0.106	0.008	0.931
Time:Ethyl_acet_vol	1	0.0	0.008	0.001	0.980
Temp:Ethyl_acet_vol	1	0.1	0.130	0.009	0.924
Pyridine_vol:Ethyl_acet_vol	1	0.0	0.007	0.000	0.983
Time:ACN_vol	1	0.0	0.014	0.001	0.975
Temp:ACN_vol	1	0.0	0.004	0.000	0.987
Pyridine_vol:ACN_vol	1	0.6	0.556	0.040	0.843
Ethyl_acet_vol:ACN_vol	1	18.5	18.519	1.328	0.258
Residuals	30	418.2	13.941		

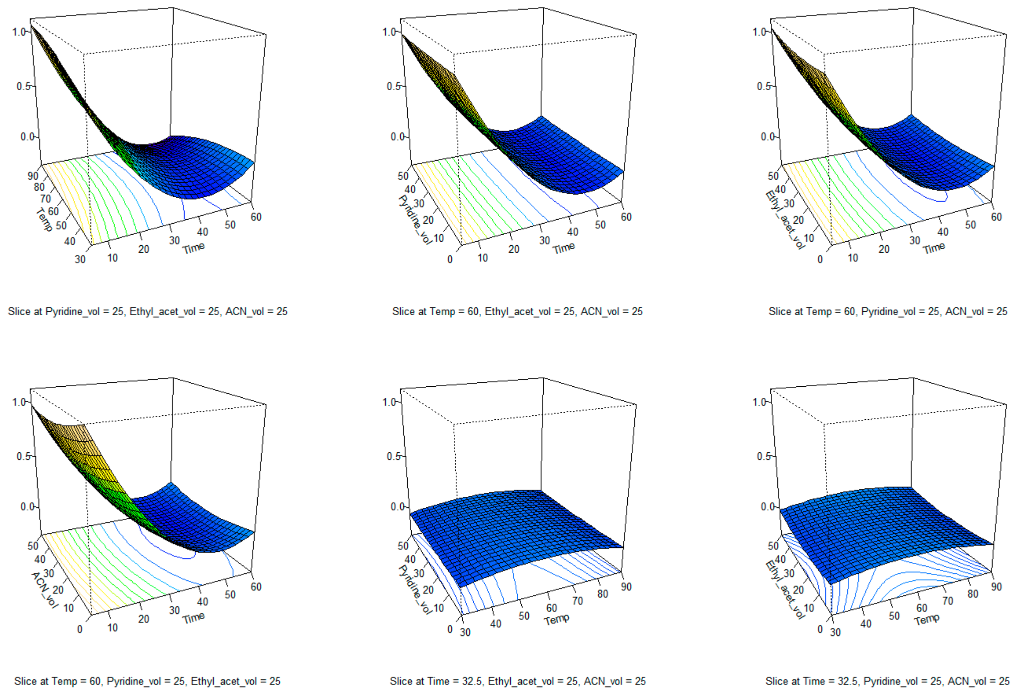

Figure S1. Response surface graphs effects on RRF of clonazepam. Reaction time has statistical significance.

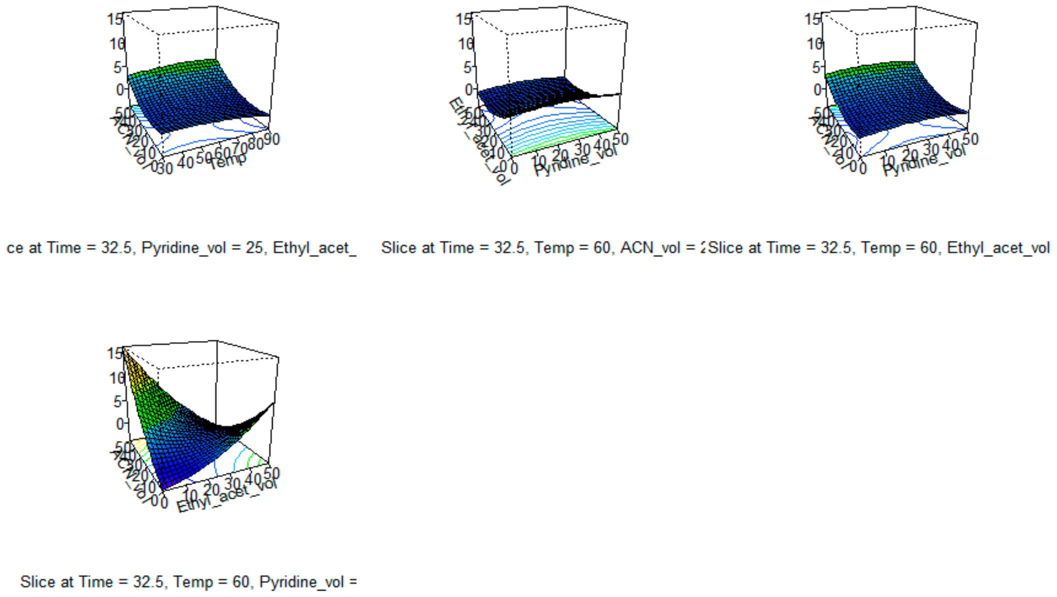

Figure S2. Response surface graphs effects on RRF of lorazepam. Interaction between acetonitrile and ethyl acetate volumes had statistical significance.

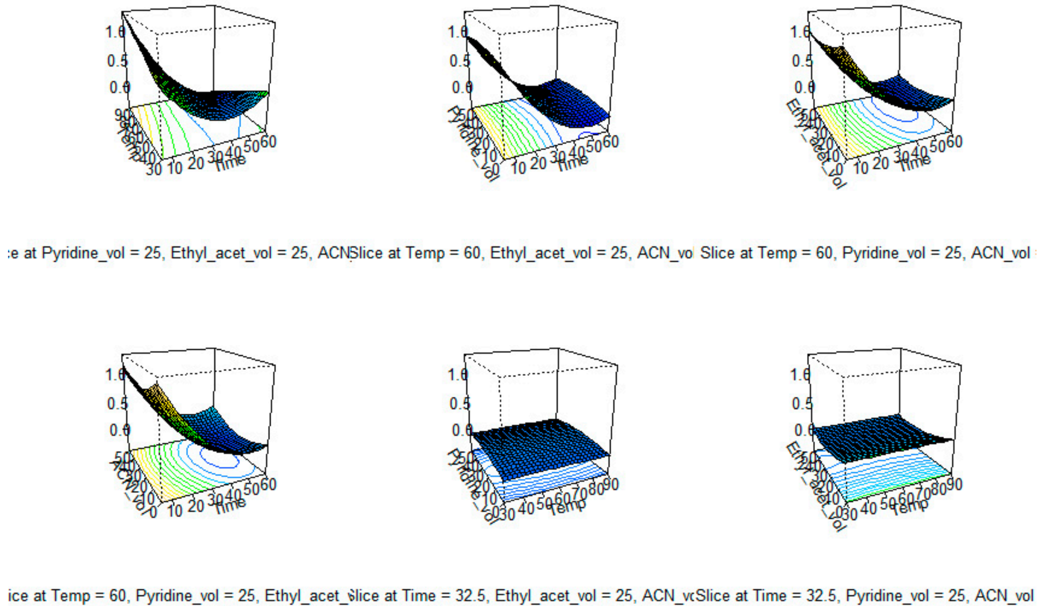

Figure S3. Response surface graphs effects on RRF of bromazepam. Reaction time has statistical significance.

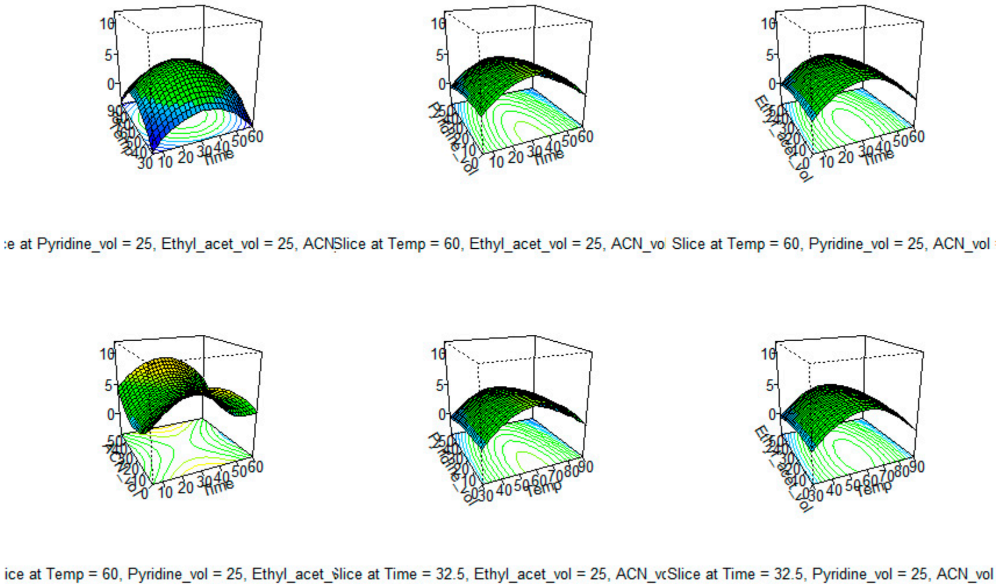

Figure S4. Response surface graphs effects on RRF of alphahydroxymidazolam. No significant effect on RRF.

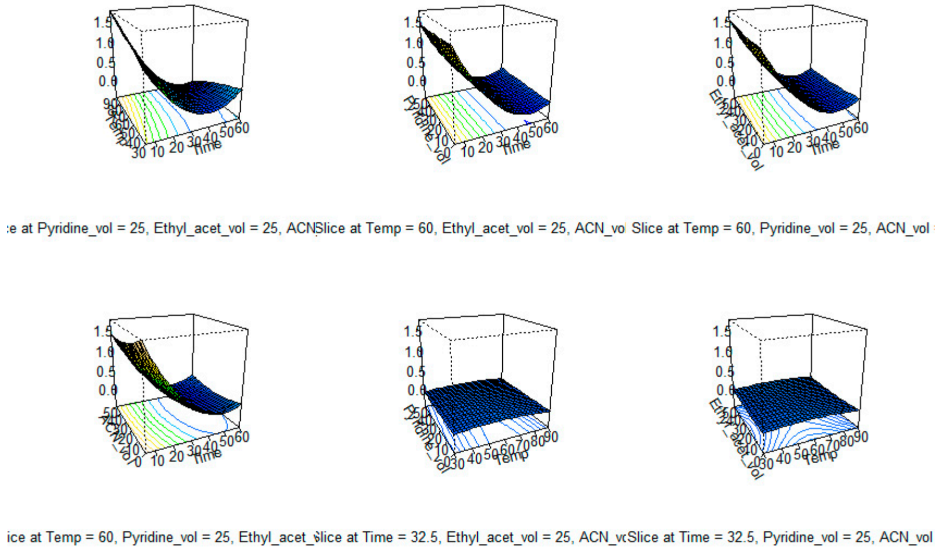

Figure S5. Response surface graphs effects on RRF of nitrazepam. Reaction time has statistical significance.
